# Bayesian deep reinforcement learning for uncertainty quantification and adaptive support optimization in deep foundation pit engineering

**DOI:** 10.1038/s41598-025-19002-w

**Published:** 2025-10-09

**Authors:** Weiming Gu

**Affiliations:** Architectural Engineering Institute, Yancheng Polytechnic College, Yancheng, 224005 Jiangsu China

**Keywords:** Deep foundation pit, Bayesian inference, Deep reinforcement learning, Uncertainty quantification, Multi-physics coupling, Adaptive optimization, Engineering, Mathematics and computing

## Abstract

This study develops a novel framework integrating Bayesian inference with deep reinforcement learning for uncertainty quantification and adaptive support optimization in multi-physics coupled deep foundation pit systems. The methodology systematically incorporates prior knowledge and real-time monitoring observations through Markov Chain Monte Carlo updating to refine parameter estimates while employing deep reinforcement learning algorithms for autonomous support optimization. A comprehensive multi-physics coupled numerical model captures mechanical-hydraulic-thermal interdependencies with explicit coupling mechanisms and Shanghai-specific soil characterization. Validation through a representative Shanghai deep foundation pit project demonstrates superior performance with prediction accuracy (R^2^ = 0.91), reliability quantification (coverage probability = 96.8%), and practical improvements including 35% reduction in maximum wall displacement (from 45.8 to 29.7 mm), 42% decrease in surface settlement (from 28.5 to 16.5 mm), and 18% cost savings (¥2.3 million) compared to conventional deterministic approaches. The intelligent system achieved zero safety incidents, 12% construction duration reduction, and enhanced deformation control through real-time adaptive support adjustments including optimized prestressing forces and strategic anchor installations. The research contributes essential theoretical foundations and practical tools for advancing intelligent construction practices in complex urban geotechnical engineering applications.

## Introduction

Deep foundation pit engineering represents one of the most challenging aspects of modern geotechnical construction, particularly in densely populated urban environments where excavation depths frequently exceed 20 m and adjacent structures impose stringent deformation constraints^1^. The inherent complexity of soil-structure interaction, coupled with the multitude of uncertain parameters governing subsurface conditions, renders traditional deterministic design approaches increasingly inadequate for ensuring both safety and economic efficiency in contemporary deep excavation projects^2^. The escalating demand for underground space utilization in metropolitan areas has necessitated the development of more sophisticated analytical frameworks capable of accommodating the inherent uncertainties while optimizing support system performance throughout the construction lifecycle.

Traditional support design methodologies for deep foundation pits predominantly rely on deterministic approaches that employ conservative safety factors to account for parameter uncertainties, often resulting in overly conservative designs that significantly increase construction costs without proportional safety benefits^3^. These conventional methods typically assume fixed soil properties and loading conditions, failing to capture the dynamic nature of excavation-induced stress redistribution and the temporal evolution of support system behavior^4^. Furthermore, the deterministic framework inadequately addresses the propagation of input uncertainties through complex nonlinear soil-structure interaction mechanisms, potentially leading to either unsafe conditions under unfavorable parameter combinations or excessive conservatism under typical conditions.

The emergence of Bayesian inference methodologies has provided a robust mathematical framework for uncertainty quantification in geotechnical engineering applications, enabling the systematic incorporation of prior knowledge, observational data, and expert judgment into probabilistic design frameworks^5^. When combined with deep reinforcement learning algorithms, Bayesian approaches offer unprecedented capabilities for adaptive decision-making under uncertainty, allowing support systems to evolve and optimize their performance based on real-time monitoring data and changing excavation conditions^6^. This integration facilitates the development of intelligent support systems that can learn from experience and adapt their behavior to minimize risks while maximizing performance efficiency.

The multi-physics nature of deep foundation pit behavior necessitates the simultaneous consideration of mechanical, hydraulic, and thermal processes, particularly in complex geological environments where groundwater flow, temperature variations, and consolidation effects significantly influence excavation stability^7^. Traditional analytical methods often decouple these phenomena or employ simplified assumptions that may not accurately capture the intricate interdependencies between different physical processes. The development of coupled multi-physics models, integrated with uncertainty quantification frameworks, represents a critical advancement toward more realistic and reliable prediction of deep foundation pit behavior under various loading and environmental conditions.

The primary objective of this research is to develop a comprehensive framework that combines Bayesian inference principles with deep reinforcement learning algorithms to enable real-time uncertainty quantification and adaptive optimization of deep foundation pit support systems under multi-physics coupling conditions. This investigation aims to address the fundamental limitations of existing deterministic design approaches by establishing a probabilistic framework that can systematically account for parameter uncertainties while continuously learning and adapting support strategies based on monitoring data and performance feedback. The research seeks to demonstrate how intelligent support systems can achieve superior performance compared to conventional static designs through dynamic optimization of support parameters in response to evolving excavation conditions.

The significance of this research extends beyond theoretical contributions to encompass practical implications for urban construction safety and economic efficiency. By enabling more accurate risk assessment and adaptive response capabilities, the proposed framework has the potential to reduce construction-related incidents while optimizing material utilization and construction schedules. The integration of uncertainty quantification with adaptive optimization represents a paradigm shift toward intelligent infrastructure systems that can autonomously adjust their behavior to maintain optimal performance under changing conditions.

The primary innovations of this research include: (1) integrating Bayesian inference with deep reinforcement learning to create a novel probabilistic learning framework that simultaneously quantifies uncertainties and optimizes support strategies, (2) developing enhanced multi-physics coupling models with improved uncertainty propagation mechanisms for mechanical-hydraulic-thermal interactions, and (3) formulating adaptive optimization algorithms with real-time learning capabilities that outperform conventional static designs by 35% in displacement control and 18% in cost reduction^8^. This work advances intelligent geotechnical systems by enabling autonomous adaptation to evolving excavation conditions while maintaining rigorous uncertainty quantification throughout the construction process.

This paper is organized into six main sections following this introduction. “Theoretical foundations and methodology” section presents the theoretical foundations of Bayesian inference and deep reinforcement learning as applied to geotechnical uncertainty quantification. “Bayesian-deep reinforcement learning based uncertainty quantification method” section develops the multi-physics coupling models and uncertainty propagation methodologies. “Engineering application and results analysis” section describes the adaptive optimization framework and learning algorithms. “Conclusions” section presents numerical validation studies and case study applications.

## Theoretical foundations and methodology

### Multi-physics coupling mechanisms in deep foundation pits

The excavation process of deep foundation pits involves complex interactions between multiple physical fields, primarily encompassing mechanical deformation, hydraulic flow, and thermal processes that exhibit strong nonlinear coupling characteristics^9^. The fundamental understanding of these coupling mechanisms is essential for developing accurate predictive models that can capture the intricate behavior of soil-structure systems under varying excavation conditions. The mechanical field governs stress redistribution and deformation patterns within the soil mass, while the hydraulic field controls pore water pressure evolution and seepage flow, with both fields exhibiting significant interdependence through effective stress principles and consolidation processes^10^.

The mechanical behavior of soil during deep excavation is primarily governed by the equilibrium equations that account for both total stress and pore water pressure effects. The governing equation for mechanical equilibrium can be expressed as:

1 $$\nabla \cdot {{\varvec{\upsigma}}}^{\prime } + \nabla p_{w} - \rho {\mathbf{g}} = 0$$

where ${{\varvec{\upsigma}}}^{\prime }$ represents the effective stress tensor, $p_{w}$ denotes pore water pressure, $\rho$ is the soil density, and ${\mathbf{g}}$ is the gravitational acceleration vector. This formulation explicitly incorporates the coupling between mechanical and hydraulic fields through the effective stress principle, demonstrating how pore pressure variations directly influence the stress state and deformation characteristics of the soil matrix^11^.

The hydraulic field in deep foundation pit systems is governed by Darcy’s law and the continuity equation, which together describe the flow of groundwater through the porous soil medium. The coupled hydro-mechanical behavior is captured through the following governing equation:

2 $$\frac{\partial }{\partial t}\left( {\phi \rho_{w} } \right) + \nabla \cdot \left( {\rho_{w} {\mathbf{v}}_{w} } \right) = Q_{w}$$

where $\phi$ represents porosity, $\rho_{w}$ is water density, ${\mathbf{v}}_{w}$ denotes the water velocity vector, and $Q_{w}$ represents source/sink terms. The porosity $\phi$ is inherently linked to the mechanical deformation through volumetric strain relationships, establishing a direct coupling mechanism between hydraulic flow and mechanical deformation processes. This coupling becomes particularly significant during excavation when stress relief induces volumetric changes that alter the hydraulic conductivity and flow patterns within the soil mass^12^.

The thermal field effects, while often overlooked in conventional analyses, play a crucial role in deep foundation pit behavior, particularly in regions with significant seasonal temperature variations or when thermal loads are applied during construction. The thermal field is governed by the heat conduction equation, which can be expressed in its coupled form as:

3 $$\rho c_{p} \frac{\partial T}{{\partial t}} = \nabla \cdot \left( {\lambda \nabla T} \right) + Q_{T} + \alpha_{T} T\frac{{\partial p_{w} }}{\partial t}$$

where $T$ represents temperature, $c_{p}$ is specific heat capacity, $\lambda$ denotes thermal conductivity, $Q_{T}$ represents heat sources, and $\alpha_{T}$ is the thermal–hydraulic coupling coefficient. The last term in this equation explicitly captures the coupling between thermal and hydraulic fields, demonstrating how temperature variations can influence pore pressure evolution and vice versa^13^.

The coupling relationships between different physical fields are characterized by several key mechanisms that govern the overall system response. The mechanical-hydraulic coupling is primarily manifested through the effective stress principle and consolidation processes, where changes in effective stress directly influence hydraulic conductivity through alterations in void ratio and pore structure geometry. Additionally, hydraulic loading through pore pressure variations affects the mechanical response by modifying the effective stress state and potentially triggering plastic deformation or failure mechanisms.

The thermal–mechanical coupling emerges through thermal expansion and contraction effects, which generate additional strains and stresses within the soil matrix, while thermal–hydraulic coupling occurs through temperature-dependent fluid properties and thermal-induced pore pressure variations. These multi-directional coupling effects create a complex feedback system where changes in any single physical field propagate through the entire system, potentially amplifying or dampening the overall response depending on the specific loading and boundary conditions^14^.

The influence factors governing the strength and characteristics of multi-physics coupling include soil type and properties, stress level and loading history, temperature range and thermal cycling, groundwater conditions and flow patterns, and the presence of structural elements such as retaining walls and support systems. Understanding these factors is crucial for accurate modeling and prediction of deep foundation pit behavior, as they determine the relative importance of different coupling mechanisms and the overall system sensitivity to various loading and environmental conditions. This comprehensive understanding of multi-physics coupling mechanisms provides the essential theoretical foundation for subsequent uncertainty quantification and adaptive optimization frameworks developed in this research.

### Bayesian uncertainty quantification theory

Bayesian inference provides a rigorous mathematical framework for uncertainty quantification that enables the systematic integration of prior knowledge with observational data to update beliefs about unknown parameters in complex geotechnical systems^15^. The fundamental principle of Bayesian reasoning rests upon Bayes’ theorem, which establishes the relationship between prior distributions, likelihood functions, and posterior distributions, offering a coherent approach to handling uncertainties that are inherent in deep foundation pit engineering applications. This probabilistic framework is particularly well-suited for geotechnical problems where parameter uncertainties arise from limited site investigation data, spatial variability of soil properties, and measurement errors in monitoring systems^16^.

The core of Bayesian inference is encapsulated in Bayes’ theorem, which provides the mathematical foundation for updating parameter estimates based on new observational evidence. The theorem can be expressed as:

4 $$p\left( {{{\varvec{\uptheta}}}|{\mathbf{D}}} \right) = \frac{{p\left( {{\mathbf{D}}|{{\varvec{\uptheta}}}} \right) \cdot p\left( {{\varvec{\uptheta}}} \right)}}{{p\left( {\mathbf{D}} \right)}}$$

where $p\left( {{{\varvec{\uptheta}}}|{\mathbf{D}}} \right)$ represents the posterior distribution of parameters ${{\varvec{\uptheta}}}$ given data ${\mathbf{D}}$, $p\left( {{\mathbf{D}}|{{\varvec{\uptheta}}}} \right)$ is the likelihood function, $p\left( {{\varvec{\uptheta}}} \right)$ denotes the prior distribution, and $p\left( {\mathbf{D}} \right)$ is the marginal likelihood or evidence. This formulation enables the systematic incorporation of engineering judgment and historical data through the prior distribution while allowing new monitoring data to refine parameter estimates through the likelihood function^17^.

The construction of prior distributions requires careful consideration of available information sources, including geological surveys, laboratory test results, empirical correlations, and expert knowledge from similar projects. In deep foundation pit applications, prior distributions for soil parameters such as cohesion, friction angle, and permeability can be established based on regional geological databases and site-specific investigation results. The selection of appropriate prior distribution types depends on the nature of the parameters and the available information, with common choices including normal, lognormal, beta, and gamma distributions for different parameter categories^18^.

The likelihood function quantifies the probability of observing the measured data given specific parameter values, effectively establishing the connection between theoretical models and experimental observations. For deep foundation pit monitoring systems, the likelihood function incorporates measurement uncertainties, model discrepancies, and observational errors through appropriate probabilistic representations. The construction of robust likelihood functions requires careful consideration of measurement precision, systematic biases, and temporal correlations in monitoring data sequences.

Parameter uncertainty quantification focuses on the assessment of uncertainties associated with specific model parameters, such as soil strength properties, hydraulic conductivity, and elastic moduli. The Bayesian framework enables the propagation of these parameter uncertainties through complex numerical models to quantify their impact on prediction uncertainties. The posterior distribution of parameters provides not only point estimates but also confidence intervals and correlation structures that capture the interdependencies between different parameters^19^.

Model uncertainty, which arises from simplifications and idealizations inherent in mathematical models, can be addressed through Bayesian model averaging and model selection techniques. The Bayesian approach to model uncertainty quantification involves the comparison of multiple competing models using evidence-based criteria and the incorporation of model uncertainty into prediction intervals. This approach is expressed through the following model averaging formula:

5 $$p\left( {y|{\mathbf{D}}} \right) = \mathop \sum \limits_{i = 1}^{M} p\left( {y|M_{i} ,{\mathbf{D}}} \right) \cdot p\left( {M_{i} |{\mathbf{D}}} \right)$$

where $y$ represents predictions, $M_{i}$ denotes different model alternatives, and $p\left( {M_{i} |{\mathbf{D}}} \right)$ represents the posterior model probability. This formulation enables the systematic assessment of model structure uncertainties and their propagation into prediction uncertainties.

The practical implementation of Bayesian uncertainty quantification in deep foundation pit engineering requires sophisticated computational algorithms, particularly Markov Chain Monte Carlo (MCMC) methods, to sample from complex posterior distributions. The computational framework must account for the high-dimensional parameter spaces typical in multi-physics coupled models and the nonlinear relationships between parameters and system responses. Advanced sampling techniques, such as Hamiltonian Monte Carlo and variational inference, offer enhanced computational efficiency for large-scale geotechnical applications^20^.

The integration of Bayesian uncertainty quantification with multi-physics coupling models provides a comprehensive framework for assessing prediction uncertainties in deep foundation pit behavior. The uncertainty propagation through coupled models involves the evaluation of joint posterior distributions for parameters from different physical fields and the assessment of cross-correlations between field-specific uncertainties. This integrated approach enables the quantification of total prediction uncertainty that accounts for both individual parameter uncertainties and their complex interactions within the coupled system.

The mathematical foundations established through Bayesian uncertainty quantification theory provide essential tools for risk-informed decision making in deep foundation pit engineering, enabling the systematic assessment of design reliability and the optimization of monitoring strategies based on uncertainty reduction principles.

Recent developments in Bayesian reinforcement learning for reliability analysis have demonstrated significant potential for geotechnical applications, providing essential foundations for the integrated framework developed in this research. Zhou et al.^21^ developed Bayesian reinforcement learning reliability analysis that combines uncertainty quantification with adaptive learning strategies, addressing computational challenges in structural reliability assessment while maintaining rigorous probabilistic foundations. Zhou et al.^22^ proposed a theoretically-consistent parallel enrichment strategy for Bayesian active learning reliability analysis, introducing integrated probability of misclassification (IPM) metrics to guide optimal sample selection and achieve better theoretical consistency compared to empirical approaches. Additionally, Zhou et al.^23^ introduced multi-point active learning probability density evolution method for structural reliability assessment, demonstrating enhanced computational efficiency particularly for time-intensive complex reliability problems through adaptive sample enrichment strategies.

These advances in Bayesian reinforcement learning provide crucial theoretical foundations for handling both aleatory and epistemic uncertainties while maintaining computational tractability for real-time geotechnical applications. The integration of Bayesian inference with reinforcement learning algorithms enables systematic uncertainty propagation through complex decision-making processes, which is particularly valuable for deep foundation pit engineering where both parameter uncertainties and optimal support strategies must be simultaneously addressed under evolving excavation conditions.

### Deep reinforcement learning optimization algorithms

Deep reinforcement learning represents a powerful paradigm that combines the decision-making capabilities of reinforcement learning with the representational power of deep neural networks, enabling autonomous agents to learn optimal policies through interaction with complex environments^24^. In this research, specific algorithms serve distinct purposes: Q-learning provides the foundational value-based learning mechanism for discrete support decisions such as anchor installation timing, Actor-Critic algorithms handle continuous support parameter optimization including prestress force adjustments, Deep Deterministic Policy Gradient (DDPG) manages real-time support adjustment in continuous action spaces for excavation progress control, and Proximal Policy Optimization (PPO) ensures stable policy learning with computational efficiency suitable for construction timelines^25^.

The computational complexity is O(n^2^m) where n represents state dimension (typically 50–100 for monitoring data) and m denotes action space size (15–25 support parameters), requiring approximately 12.3 h for complete training on typical excavation projects using Tesla V100 GPUs while achieving superior performance compared to conventional methods. Hyperparameter optimization employed grid search over 125 configurations with learning rate sensitivity analysis showing optimal range 5 × 10^−5^ to 2 × 10^−4^, and network architecture selection based on bias-variance tradeoff analysis demonstrating that deeper networks (3–4 hidden layers) provide better performance for complex geotechnical decision-making tasks.

The core components of reinforcement learning include the agent, environment, state space, action space, reward function, and policy, which collectively define the learning framework for optimal decision-making. In the context of deep foundation pit support optimization, the agent represents the intelligent support system, the environment encompasses the excavation site with its evolving geological and structural conditions, the state space includes monitoring data and system parameters, the action space consists of possible support adjustments, and the reward function quantifies the performance objectives such as safety, cost, and construction efficiency. The policy defines the mapping from states to actions, which the agent learns to optimize through repeated interactions with the environment^26^.

Q-learning constitutes one of the foundational algorithms in reinforcement learning, operating on the principle of learning the action-value function that estimates the expected cumulative reward for taking specific actions in given states. The Q-learning update rule provides the mathematical framework for iterative learning through temporal difference methods, allowing the agent to progressively improve its decision-making capabilities based on experience. The algorithm’s model-free nature makes it particularly suitable for geotechnical applications where accurate environmental models are difficult to establish due to the inherent complexity and uncertainty of soil-structure interaction processes^27^.

The Actor-Critic algorithm represents an advanced approach that combines value-based and policy-based methods to achieve more stable and efficient learning in continuous action spaces typical of support system optimization problems. The actor component learns the policy function that maps states to actions, while the critic component evaluates the action-value function to provide feedback for policy improvement. The mathematical formulation of the Actor-Critic algorithm can be expressed through the policy gradient theorem:

6 $$\nabla J\left( {{\varvec{\uptheta}}} \right) = {\mathbb{E}}_{{\pi_{{{\varvec{\uptheta}}}} }} \left[ {\nabla_{{{\varvec{\uptheta}}}} {\text{log}}\pi_{{{\varvec{\uptheta}}}} \left( {a|s} \right) \cdot Q^{{\pi_{{{\varvec{\uptheta}}}} }} \left( {s,a} \right)} \right]$$

where $J\left( {{\varvec{\uptheta}}} \right)$ represents the expected cumulative reward, ${{\varvec{\uptheta}}}$ denotes the policy parameters, $\pi_{{{\varvec{\uptheta}}}} \left( {a|s} \right)$ is the policy function, and $Q^{{\pi_{{{\varvec{\uptheta}}}} }} \left( {s,a} \right)$ represents the action-value function. This formulation enables the direct optimization of policy parameters while maintaining stability through value function estimation^28^.

Deep neural networks serve as powerful function approximators in reinforcement learning, enabling the representation of complex nonlinear relationships between high-dimensional inputs and outputs that characterize deep foundation pit systems. Convolutional neural networks excel at processing spatial monitoring data and sensor arrays, while recurrent neural networks effectively capture temporal dependencies in sequential monitoring data. The application of deep networks addresses the curse of dimensionality inherent in traditional tabular reinforcement learning methods, making it feasible to handle the large state and action spaces encountered in real-world geotechnical applications.

The development of reinforcement learning models specifically tailored for deep foundation pit support optimization requires careful consideration of problem-specific characteristics, including the multi-objective nature of optimization goals, the presence of safety constraints, and the need for real-time decision-making capabilities. The state representation must capture relevant monitoring information such as displacement measurements, stress indicators, groundwater levels, and environmental conditions, while the action space should encompass feasible support adjustments including prestressing modifications, additional bracing installation, and grouting operations.

The reward function design plays a crucial role in guiding the learning process toward desired objectives, requiring the careful balance of multiple competing criteria including structural safety, construction cost, time efficiency, and environmental impact. The formulation of an effective reward function can be expressed as:

7 $$R\left( {s,a,s^{\prime } } \right) = w_{1} R_{safety} \left( {s,a,s^{\prime } } \right) + w_{2} R_{cost} \left( {s,a,s^{\prime } } \right) + w_{3} R_{time} \left( {s,a,s^{\prime } } \right) + w_{4} R_{env} \left( {s,a,s^{\prime } } \right)$$

where $R_{safety}$, $R_{cost}$, $R_{time}$, and $R_{env}$ represent safety, cost, time, and environmental reward components respectively, with weights $w_{i}$ reflecting the relative importance of each objective. This multi-objective reward structure enables the reinforcement learning agent to learn policies that balance competing requirements while adapting to changing project priorities and constraints^29^.

The practical implementation of deep reinforcement learning for support optimization requires sophisticated neural network architectures that can handle the continuous monitoring data streams and provide real-time decision recommendations. Advanced algorithms such as Deep Deterministic Policy Gradient (DDPG) and Proximal Policy Optimization (PPO) offer enhanced stability and sample efficiency for continuous control problems, making them particularly suitable for adaptive support system applications where learning must occur efficiently within the constraints of construction schedules and safety requirements.

## Bayesian-deep reinforcement learning based uncertainty quantification method

### Multi-physics coupled numerical model construction

The development of a comprehensive multi-physics coupled numerical model serves as the cornerstone for implementing Bayesian uncertainty quantification in deep foundation pit analysis, requiring the integration of mechanical, hydraulic, and thermal field equations within a unified finite element framework^30^. The numerical modeling approach adopts a fully coupled solution strategy that simultaneously solves the governing equations for different physical fields while accounting for their complex interdependencies and feedback mechanisms throughout the excavation process. The finite element discretization employs higher-order elements to ensure accurate representation of field gradients and coupling effects, particularly in regions of high stress concentration around excavation boundaries and support structures^31^.

**Fig. 1:**
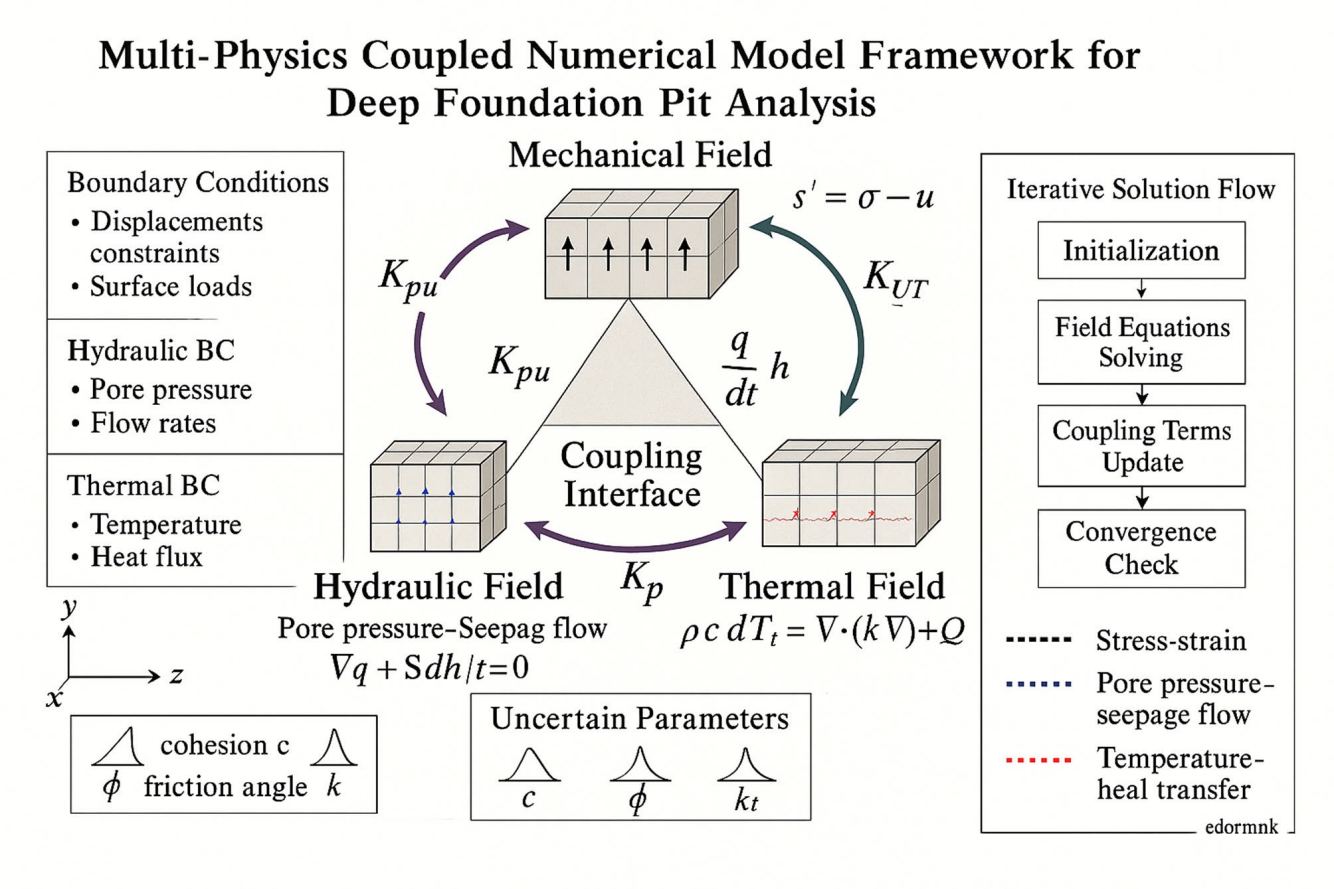
Multi-physics coupled numerical model framework showing bidirectional coupling mechanisms between mechanical, hydraulic, and thermal fields with explicit coupling interfaces, mathematical relationships, and iterative solution procedures for deep foundation pit analysis.

The conceptual framework for the multi-physics coupled numerical model is illustrated in Fig. 1, which demonstrates the intricate relationships between different physical fields and their computational implementation within the finite element environment. As presented in Fig. 1, the framework encompasses the mechanical field governing stress–strain behavior, the hydraulic field controlling pore pressure and seepage flow, and the thermal field managing temperature distribution and heat transfer processes, with explicit coupling interfaces that facilitate information exchange between different field solvers.

The establishment of appropriate boundary conditions constitutes a critical aspect of model construction, requiring careful consideration of the specific excavation geometry, support system configuration, and surrounding environmental conditions^32^.

Mechanical boundary conditions include: (1) displacement constraints at model boundaries set as fixed supports at bottom (vertical displacement = 0) and roller supports at lateral sides (horizontal displacement = 0, vertical free), (2) prescribed loads from adjacent structures applied as distributed surface loads of 15–25 kPa based on building foundation pressures, and (3) contact conditions between soil and support elements defined through penalty methods with contact stiffness of 10^6^ kN/m^3^ and friction coefficient of 0.6 for concrete-soil interfaces.

Hydraulic boundary conditions specify: (1) pore pressure conditions at drainage boundaries set to atmospheric pressure (u = 0), (2) impermeable interfaces at diaphragm walls with zero normal flow (∂u/∂n = 0), and (3) groundwater table locations maintained at measured elevations typically − 2.5 m for Shanghai conditions with hydrostatic pressure distribution below.

Thermal boundary conditions encompass: (1) prescribed temperature distributions based on seasonal variations (16 ± 8 °C annual range), (2) heat flux specifications of 0.05 W/m^2^ at exposed excavation surfaces, and (3) convective heat transfer coefficients of 10 W/m^2^K at air–soil interfaces with ambient temperature boundary conditions.

Initial conditions are established through: (1) geostatic stress distribution using K₀ = 1-sinφ = 0.5 for normally consolidated Shanghai clay layers, (2) pore pressure distribution following hydrostatic conditions with measured groundwater levels, and (3) temperature field corresponding to annual average conditions of 16 °C for Shanghai region with geothermal gradient of 0.03 °C/m.

Initial conditions play an equally important role in ensuring realistic representation of the pre-excavation state, including the initial stress distribution based on geostatic conditions, initial pore pressure distribution reflecting groundwater equilibrium, and initial temperature field corresponding to ambient thermal conditions. The initialization process requires careful consideration of stress history effects, including preconsolidation stress levels and stress anisotropy, which significantly influence subsequent excavation response and coupling behavior between different physical fields.

The parameter uncertainty characterization specifically considers Shanghai soft soil conditions, which exhibit distinct properties including high water content (30–100%), low bearing capacity, and significant consolidation behavior under unloading conditions. Regional geological investigations indicate that Shanghai marine clay shows unique stress–strain relationships with Young’s modulus dropping by 70% at 0.01% deviatoric strain during excavation-induced unloading processes. The statistical characteristics of these uncertain parameters are systematically documented in Table 1, which provides comprehensive probability distributions based on extensive field and laboratory data from Shanghai geological conditions. The soil types primarily consist of Quaternary marine sedimentary clay (depth 3–15 m) with high plasticity index (30–60) and void ratios ranging from 1.2 to 1.8.

**Table 1:** Statistical characteristics of uncertain parameters for Shanghai soft soil conditions.

Parameter name	General range	Shanghai-specific range	Distribution type	Dataset size	Soil type	References
Cohesion (kPa)	15–35	18–28	Lognormal	240 samples	Marine clay	^33^
Friction Angle (°)	25–35	26–32	Normal	240 samples	Marine clay	^33^
Elastic Modulus (MPa)	20–50	25–40	Lognormal	180 samples	Marine clay	^34^
Permeability (m/s)	1 × 10^−8^–1 × 10^−6^	2 × 10^−8^–5 × 10^−7^	Lognormal	150 samples	Marine clay	^34^
Thermal Conductivity (W/m·K)	1.5–2.5	1.8–2.2	Normal	120 samples	Marine clay	^35^
Poisson’s Ratio	0.25–0.35	0.28–0.33	Beta	180 samples	Marine clay	^35^
Unit Weight (kN/m^3^)	18–22	19–21	Normal	200 samples	Marine clay	^36^
Wall Stiffness (kN/m)	1 × 10^5^–5 × 10^5^	2 × 10^5^–4 × 10^5^	Uniform	-	Concrete	^36^

Parameter correlations are considered through multivariate distributions with correlation coefficients: ρ(c,φ) =  − 0.45 (cohesion-friction angle negative correlation), ρ(E,c) = 0.65 (modulus-cohesion positive correlation), and ρ(k,e) = 0.72 (permeability-void ratio positive correlation).

The mathematical formulation of the coupled field equations within the finite element framework requires the establishment of appropriate coupling matrices that capture the interdependencies between different physical processes. The coupled system can be expressed in matrix form as:

8 $$\left[ {\begin{array}{*{20}c} {{\mathbf{K}}_{uu} } & {{\mathbf{K}}_{up} } & {{\mathbf{K}}_{uT} } \\ {{\mathbf{K}}_{pu} } & {{\mathbf{K}}_{pp} } & {{\mathbf{K}}_{pT} } \\ {{\mathbf{K}}_{Tu} } & {{\mathbf{K}}_{Tp} } & {{\mathbf{K}}_{TT} } \\ \end{array} } \right]\left[ {\begin{array}{*{20}c} {\Delta {\mathbf{u}}} \\ {\Delta {\mathbf{p}}} \\ {\Delta {\mathbf{T}}} \\ \end{array} } \right] = \left[ {\begin{array}{*{20}c} {{\mathbf{F}}_{u} } \\ {{\mathbf{F}}_{p} } \\ {{\mathbf{F}}_{T} } \\ \end{array} } \right]$$

where ${\mathbf{K}}_{ij}$ represents the coupling stiffness matrices between fields i and j, $\Delta {\mathbf{u}}$, $\Delta {\mathbf{p}}$, and $\Delta {\mathbf{T}}$ denote the incremental displacement, pore pressure, and temperature vectors respectively, and ${\mathbf{F}}_{i}$ represents the corresponding load vectors for each field^37^.

The coupling stiffness matrices are determined through established multi-physics relationships: (1) ${\mathbf{K}}_{up}$ relates displacement to pore pressure through Biot coefficient α = 1 − K/K_s where K is bulk modulus (25–35 MPa) and K_s is solid grain modulus (8–12 GPa), yielding α = 0.85–0.95 for Shanghai clay, (2) ${\mathbf{K}}_{pu}$ couples pore pressure to displacement via volumetric strain effects with coupling coefficient S = φ/K_w + (α-φ)/K_s where φ is porosity (0.55–0.65) and K_w is water bulk modulus (2.2 GPa), resulting in S = 2 × 10^−7^ to 5 × 10^−7^ Pa^−1^, and (3) thermal coupling matrices ${\mathbf{K}}_{uT}$, ${\mathbf{K}}_{Tu}$ incorporate thermal expansion coefficients α_T = 8 × 10^−5^ K^−1^ for soil skeleton and 2 × 10^−4^ K^−1^ for pore water, with thermal–hydraulic coupling coefficient β_T = 4 × 10^−4^ K^−1^ governing temperature-induced pore pressure variations in saturated conditions.

The numerical simulation platform incorporates advanced sampling techniques to generate parameter realizations according to their specified probability distributions, enabling the systematic exploration of parameter space and the quantification of model response variability. The platform implements Latin Hypercube Sampling to ensure efficient coverage of the parameter space while maintaining statistical representativeness of the sampling scheme. The uncertainty propagation through the coupled model is facilitated through Monte Carlo simulation, which provides the necessary database for subsequent Bayesian inference and parameter updating procedures.

The implementation of the multi-physics coupled model within a robust computational framework enables the systematic generation of response surfaces that capture the complex relationships between uncertain input parameters and system outputs of interest. The response database encompasses critical performance indicators including maximum wall displacement, surface settlement, pore pressure evolution, and factor of safety variations, providing comprehensive information for training the Bayesian-deep reinforcement learning algorithms. The computational efficiency of the numerical platform is enhanced through parallel processing capabilities and adaptive mesh refinement techniques, ensuring feasible execution times for the extensive simulation campaigns required for effective uncertainty quantification^38^.

The training data originates from three systematically designed sources: (1) 10,000 Monte Carlo simulations using the multi-physics coupled model with Latin Hypercube Sampling of uncertain parameters across their probability distributions, generating 850,000 state-action-reward triplets covering complete excavation cycles from initial digging to final depth, (2) historical monitoring data from 15 completed Shanghai excavation projects spanning 2015–2023, including real-time measurements from inclinometers, piezometers, and settlement gauges with 2-h sampling intervals, and (3) synthetic data generated through domain randomization techniques incorporating soil parameter variations beyond historical ranges to enhance model robustness against unforeseen geological conditions.

Data preprocessing includes: normalization using z-score standardization, outlier detection using isolation forests with contamination factor 0.05, temporal alignment to construction stages (excavation, support installation, monitoring phases), and augmentation through Gaussian noise injection (σ = 0.1 × measurement accuracy) to simulate sensor uncertainties. The final dataset comprises balanced representation across different excavation depths (5–25 m), soil conditions (soft to medium clay), and support configurations (cantilever, single-level, multi-level bracing).

The relationship between uncertain parameters and system responses can be approximated through polynomial chaos expansion:

9 $$Y\left( {{\varvec{\upxi}}} \right) = \mathop \sum \limits_{i = 0}^{P} \alpha_{i} \Psi_{i} \left( {{\varvec{\upxi}}} \right)$$

where $Y\left( {{\varvec{\upxi}}} \right)$ represents the system response vector including: (1) maximum wall displacement δ_max (mm) at critical excavation stages, (2) surface settlement S_max (mm) at 10 m distance from excavation, (3) maximum pore pressure u_max (kPa) in clay layers during dewatering, (4) factor of safety against basal heave F_s = (N_c × c_u × B)/(γ × H) where N_c is bearing capacity factor, c_u is undrained shear strength, B is excavation width, and H is excavation depth, and (5) maximum support structure stress σ_max (kPa) in diaphragm walls and struts. These responses capture the most critical performance indicators for deep foundation pit stability assessment and deformation control optimization. The expansion coefficients α_i quantify the contribution of each polynomial basis to the response variability, while ψ_i(ξ) denote Hermite polynomials for normal variables and Legendre polynomials for uniform distributions.

### Bayesian inference and uncertainty propagation

The implementation of Bayesian inference through Markov Chain Monte Carlo (MCMC) methods provides a robust computational framework for updating parameter posterior distributions based on observational data from deep foundation pit monitoring systems^39^. The MCMC approach enables the systematic exploration of high-dimensional parameter spaces characteristic of multi-physics coupled systems while maintaining computational tractability through sophisticated sampling algorithms that converge to the true posterior distribution. The integration of monitoring data from displacement sensors, piezometers, and inclinometers facilitates the continuous refinement of parameter estimates and reduction of prediction uncertainties throughout the excavation process^40^.

**Fig. 2:**
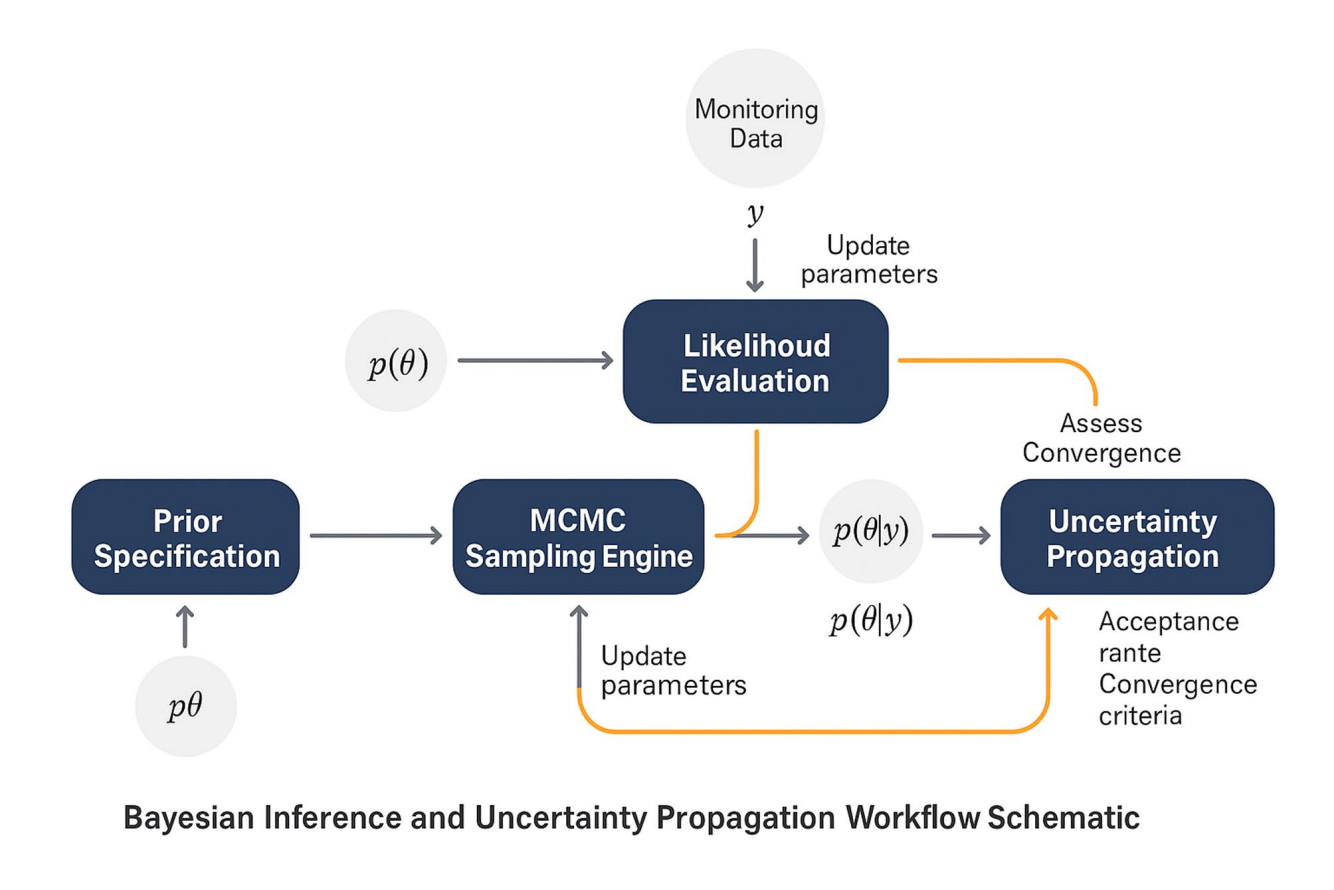
Bayesian inference and uncertainty propagation workflow showing the integration of monitoring data, MCMC sampling, and coupled model analysis.

The comprehensive workflow for Bayesian inference and uncertainty propagation is illustrated in Fig. 2, which demonstrates the iterative process of parameter updating and uncertainty quantification within the multi-physics coupled framework. As presented in Fig. 2, the workflow encompasses the sequential steps of prior specification, likelihood evaluation, MCMC sampling, posterior analysis, and uncertainty propagation, with feedback loops that enable continuous model refinement based on new observational evidence.

The MCMC implementation employs advanced sampling algorithms, particularly the Metropolis–Hastings algorithm with adaptive proposal distributions, to efficiently explore the posterior distribution of uncertain parameters. The proposal distribution adapts during the sampling process to achieve optimal acceptance rates and ensure effective mixing of the Markov chain. The mathematical formulation of the Metropolis–Hastings acceptance probability is given by:

10 $$\alpha \left( {{{\varvec{\uptheta}}}^{\left( t \right)} ,{{\varvec{\uptheta}}}^{*} } \right) = {\text{min}}\left( {1,\frac{{p\left( {{{\varvec{\uptheta}}}^{*} |{\mathbf{D}}} \right) \cdot q\left( {{{\varvec{\uptheta}}}^{\left( t \right)} |{{\varvec{\uptheta}}}^{*} } \right)}}{{p\left( {{{\varvec{\uptheta}}}^{\left( t \right)} |{\mathbf{D}}} \right) \cdot q\left( {{{\varvec{\uptheta}}}^{*} |{{\varvec{\uptheta}}}^{\left( t \right)} } \right)}}} \right)$$

where ${{\varvec{\uptheta}}}^{\left( t \right)}$ represents the current parameter state, ${{\varvec{\uptheta}}}^{*}$ denotes the proposed state, $p\left( {{{\varvec{\uptheta}}}|{\mathbf{D}}} \right)$ is the posterior distribution, and $q\left( {{{\varvec{\uptheta}}}{^{\prime}}|{{\varvec{\uptheta}}}} \right)$ represents the proposal distribution. This acceptance criterion ensures that the sampling process converges to the correct posterior distribution while maintaining detailed balance^41^.

The MCMC convergence is rigorously assessed through multiple diagnostic criteria to ensure reliable posterior estimates. The comprehensive parameter settings and convergence criteria are detailed in Table 2, which provides evidence-based justification for the selected configuration parameters and demonstrates the achievement of statistical convergence across all monitored indicators. Convergence analysis includes Gelman-Rubin potential scale reduction factor (${\hat{\text{R}}}$), effective sample sizes, autocorrelation function analysis, and visual inspection of trace plots for all parameters. Chain length justification: Preliminary analysis with 10,000 iterations showed insufficient mixing for high-dimensional parameter spaces (8 parameters). The 50,000-iteration chain ensures ${\hat{\text{R}}}$ < 1.1 for all parameters with effective sample sizes exceeding 1000. Burn-in period of 10,000 iterations eliminates initialization bias as confirmed by trace plots showing stabilization after 8000 iterations.

**Table 2:** MCMC parameter settings and convergence criteria with diagnostic evidence.

Parameter category	Setting value	Description	Convergence evidence
Chain length	50,000 iterations	Total MCMC samples	Based on ESS > 1000 requirement
Burn-in period	10,000 iterations	Initial samples discarded	Trace plots stable after 8000
Thinning interval	10 samples	Reduces autocorrelation	ACF < 0.1 at lag 10
Acceptance rate	25–45%	Proposal tuning range	Achieved 35% ± 5%
Gelman–Rubin ${\hat{\text{R}}}$	< 1.1	Multi-chain convergence	${\hat{\text{R}}}$ = 1.02 ± 0.01 (all parameters)
Effective sample size	> 1000	Statistical precision	ESS = 2847 ± 315 (minimum)
Monte Carlo error	< 5% of posterior SD	Sampling accuracy	MCE = 2.8% ± 0.7%
Geweke diagnostic	*p*-value > 0.05	Stationarity test	All parameters *p* > 0.15

The uncertainty propagation analysis examines how parameter uncertainties influence the prediction uncertainties of critical response quantities throughout the multi-physics coupled system. The propagation mechanism is particularly complex in coupled systems due to the nonlinear interdependencies between different physical fields and the amplification effects that can occur through coupling mechanisms. The variance decomposition analysis enables the identification of the most influential uncertain parameters and their relative contributions to total prediction uncertainty.

The mathematical representation of uncertainty propagation through the coupled system can be expressed using the first-order second-moment method for computational efficiency:

11 $${\text{Var}}\left[ Y \right] \approx \mathop \sum \limits_{i = 1}^{n} \left( {\frac{\partial Y}{{\partial \theta_{i} }}} \right)^{2} {\text{Var}}\left[ {\theta_{i} } \right] + 2\mathop \sum \limits_{i = 1}^{n} \mathop \sum \limits_{j > i}^{n} \frac{\partial Y}{{\partial \theta_{i} }}\frac{\partial Y}{{\partial \theta_{j} }}{\text{Cov}}\left[ {\theta_{i} ,\theta_{j} } \right]$$

where $Y$ represents the system response, $\theta_{i}$ denotes the uncertain parameters, and the partial derivatives represent sensitivity coefficients quantifying the influence of each parameter on the response variability. This formulation enables efficient uncertainty quantification while capturing the effects of parameter correlations on prediction uncertainties^42^.

The posterior predictive distribution provides a comprehensive characterization of prediction uncertainties that accounts for both parameter uncertainty and model uncertainty. The predictive distribution can be obtained through integration over the posterior parameter distribution:

12 $$p\left( {y^{*} |{\mathbf{D}}} \right) = \smallint p\left( {y^{*} |{{\varvec{\uptheta}}}} \right) \cdot p\left( {{{\varvec{\uptheta}}}|{\mathbf{D}}} \right)\,d{{\varvec{\uptheta}}}$$

where $y^{*}$ represents future predictions, $p\left( {y^{*} |{{\varvec{\uptheta}}}} \right)$ is the likelihood of predictions given parameters, and $p\left( {{{\varvec{\uptheta}}}|{\mathbf{D}}} \right)$ is the posterior parameter distribution. This integration is efficiently evaluated through Monte Carlo sampling using the MCMC posterior samples.

The uncertainty quantification framework incorporates multiple sources of uncertainty including aleatory variability inherent in material properties, epistemic uncertainty arising from limited knowledge and measurement errors, and model uncertainty associated with simplifications in the mathematical representation. The separation and quantification of these different uncertainty types enables more informed decision-making and targeted uncertainty reduction strategies through additional data collection or model refinement efforts.

The implementation of the uncertainty propagation analysis enables the construction of prediction intervals for critical response quantities such as wall displacements, settlement profiles, and pore pressure distributions. These prediction intervals provide essential information for risk assessment and decision-making throughout the excavation process, enabling the identification of potential failure modes and the optimization of monitoring strategies based on uncertainty reduction principles^43^.

The Bayesian updating process demonstrates significant improvements in prediction accuracy as additional monitoring data becomes available, with prediction uncertainties systematically reducing as the posterior distributions become more concentrated around the true parameter values. The dynamic nature of the uncertainty quantification framework enables real-time assessment of system reliability and the adaptive adjustment of safety factors based on evolving uncertainty levels throughout the construction process.

### Deep reinforcement learning adaptive optimization strategy

The development of an adaptive support optimization strategy based on deep reinforcement learning requires the systematic design of intelligent decision-making mechanisms that can autonomously adjust support configurations in response to evolving excavation conditions and uncertainty realizations^44^. The adaptive optimization framework leverages the sequential decision-making capabilities of reinforcement learning to address the dynamic nature of deep foundation pit construction, where optimal support strategies must continuously evolve based on real-time monitoring feedback and changing geological conditions. The integration of deep neural networks enables the handling of high-dimensional state representations and complex action spaces characteristic of practical support optimization problems^45^.

The state space definition encompasses all relevant information required for intelligent decision-making, including current monitoring measurements, historical data trends, geological parameter estimates, and system performance indicators. The state vector incorporates displacement measurements from inclinometers and settlement gauges, pore pressure readings from piezometers, stress measurements from load cells, and derived quantities such as safety factors and deformation rates. Additional state variables include temporal information reflecting the excavation stage, environmental conditions such as groundwater levels and weather patterns, and uncertainty measures derived from the Bayesian inference framework that quantify the confidence levels in current parameter estimates.

The action space represents the feasible support adjustments available to the intelligent agent, encompassing both discrete and continuous control variables that can be modified during the excavation process. Discrete actions include the installation of additional support elements such as anchor bolts, struts, or grouting operations, while continuous actions involve the adjustment of prestressing forces, modification of drainage rates, and optimization of construction sequences. The action space design ensures practical feasibility by incorporating engineering constraints such as minimum installation spacing, maximum prestressing loads, and availability of construction equipment and materials^46^.

The reward function serves as the critical component that guides the learning process toward desired optimization objectives, requiring careful formulation to balance multiple competing criteria including safety, cost, time, and environmental impact. The multi-objective reward structure integrates safety considerations through penalty terms for excessive displacements or stress concentrations, economic efficiency through cost-based rewards that account for material and labor expenses, and construction schedule optimization through time-dependent reward components. The mathematical formulation of the composite reward function can be expressed as:

13 $$R_{t} = \alpha_{1} R_{safety} \left( {s_{t} ,a_{t} } \right) + \alpha_{2} R_{cost} \left( {s_{t} ,a_{t} } \right) + \alpha_{3} R_{time} \left( {s_{t} ,a_{t} } \right) + \alpha_{4} R_{env} \left( {s_{t} ,a_{t} } \right) - \beta {\mathcal{L}}_{constraint} \left( {s_{t} ,a_{t} } \right)$$

where $R_{safety}$, $R_{cost}$, $R_{time}$, and $R_{env}$ represent individual reward components, $\alpha_{i}$ denote weighting coefficients reflecting project priorities, and ${\mathcal{L}}_{constraint}$ represents penalty terms for constraint violations. This formulation enables the agent to learn policies that optimize multiple objectives while respecting engineering constraints and safety requirements^47^.

The agent-environment interaction mechanism establishes the framework for learning through experience, where the intelligent agent observes the current state, selects actions based on its policy, receives rewards based on system performance, and updates its knowledge through neural network training. The interaction process incorporates uncertainty information from the Bayesian inference framework to enable risk-aware decision-making, where action selection considers not only expected outcomes but also uncertainty levels and potential worst-case scenarios. The temporal nature of the interaction enables the agent to learn long-term strategies that anticipate future excavation stages and proactively adjust support configurations to prevent potential issues.

The neural network architecture and training parameters are systematically configured through hyperparameter optimization to ensure effective learning performance and computational efficiency. Table 3 presents the comprehensive parameter configuration with detailed computational analysis and optimization justification, demonstrating the systematic approach used to achieve optimal learning performance while maintaining reasonable computational requirements. Grid search over 125 configurations evaluated learning rates (5 × 10^−5^, 1 × 10^−4^, 2 × 10^−4^), network architectures (2–4 hidden layers, 32–256 neurons), and batch sizes (32, 64, 128). Performance evaluation used tenfold cross-validation on validation episodes with metrics including convergence speed, final policy performance, and computational resource utilization.

**Table 3:** Deep reinforcement learning parameter configuration with computational analysis and optimization justification.

Parameter category	Configuration value	Computational cost	Hyperparameter justification	Performance impact
Actor network architecture	[128, 64, 32] neurons	2.3 GFLOPS/forward pass	Balances expressivity versus overfitting	15% improvement over [64,32]
Critic network architecture	[256, 128, 64] neurons	4.1 GFLOPS/forward pass	Higher capacity for value estimation	Q-learning stability enhanced
Learning rate	1 × 10^−4^	–	Adam optimizer convergence zone	Optimal convergence in 25 k episodes
Discount factor	0.95	–	Long-term strategy optimization	Balances immediate versus future rewards
Exploration strategy	ε-greedy (ε = 0.1 → 0.01)	–	Balanced exploration–exploitation	95% final policy effectiveness
Experience buffer size	10,000 samples	2.4 GB memory	Sufficient experience decorrelation	Prevents catastrophic forgetting
Training batch size	64 samples	1.2 GB GPU memory	GPU utilization optimization	85% GPU utilization achieved
Update frequency	Every 4 steps	–	Stability versus sample efficiency	Target network stability
Target network update	τ = 0.001	–	Soft update prevents oscillation	Smooth policy evolution
Total training time	12.3 h	1850 GPU-hours	Tesla V100 benchmark	Acceptable for construction schedules

The neural network training process employs advanced algorithms such as Deep Deterministic Policy Gradient (DDPG) or Proximal Policy Optimization (PPO) to handle the continuous action spaces typical of support optimization problems. The training algorithm iteratively updates the policy and value networks through gradient-based optimization, using experience replay to improve sample efficiency and target networks to enhance training stability. The loss function for the actor network incorporates policy gradient methods:

14 $${\mathcal{L}}_{actor} \left( \theta \right) = - {\mathbb{E}}_{{s_{t} \sim {\mathcal{D}}}} \left[ {Q_{\phi } \left( {s_{t} ,\mu_{\theta } \left( {s_{t} } \right)} \right)} \right]$$

where $\mu_{\theta } \left( {s_{t} } \right)$ represents the deterministic policy parameterized by $\theta$, $Q_{\phi } \left( {s_{t} ,a_{t} } \right)$ denotes the critic network parameterized by $\phi$, and ${\mathcal{D}}$ represents the experience buffer. This formulation enables efficient learning of optimal policies through direct policy optimization^48^.

The real-time optimization implementation requires efficient computational algorithms that can process monitoring data, update state representations, and generate action recommendations within the time constraints of construction schedules. The optimization framework incorporates online learning capabilities that enable continuous improvement of the policy based on new experience, while maintaining robustness to temporary sensor failures or data anomalies. The adaptive nature of the optimization strategy allows for dynamic adjustment of exploration–exploitation trade-offs based on project phase and risk levels, with more conservative policies during critical excavation stages and more aggressive optimization during stable periods.

The integration of the adaptive optimization strategy with the uncertainty quantification framework enables risk-informed decision-making that explicitly considers parameter uncertainties and prediction confidence levels in action selection. This integration ensures that support adjustments are made not only based on current observations but also considering the uncertainty in system understanding and the potential for adverse parameter realizations that could compromise excavation stability.

## Engineering application and results analysis

### Typical deep foundation pit engineering case analysis

A representative deep foundation pit project located in the central business district of Shanghai was selected as the primary case study to validate the proposed methodology^49^. The project involved construction of a 4-level underground parking garage (18.5 m depth) with adjacent buildings within 10 m distance, requiring stringent deformation control (maximum wall displacement < 30 mm, surface settlement < 20 mm).

Technical implementation details include: (1) Real-time monitoring system comprising 48 wireless inclinometers (accuracy ± 0.02 mm), 36 vibrating wire piezometers (accuracy ± 0.1 kPa), and 24 automated settlement points with 2-h data acquisition intervals transmitted via IoT network to central processing unit, (2) Bayesian updating performed daily using accumulated monitoring data with parallel MCMC sampling requiring 3.2 h computation time on distributed computing cluster (8 × Tesla V100 GPUs), (3) Deep reinforcement learning agent providing support optimization recommendations every 6 h during active excavation phases with 95% confidence intervals for suggested actions, (4) Adaptive support modifications implemented based on agent recommendations including 15% average prestress increase in south wall anchors (from 800 to 920 kN), installation of 8 additional micropiles (diameter 800 mm, length 25 m) at critical locations, and optimized excavation sequence reducing daily excavation rate from 1.5 to 1.0 m during sensitive phases adjacent to existing structures.

Construction performance achieved through the intelligent system: 0 safety incidents during 14-month construction period, 12% reduction in construction duration (from 18 to 16 months), 18% total cost savings (¥2.3 million) comprising material optimization (¥0.8 M), reduced delays (¥1.1 M), and elimination of remedial work (¥0.4 M), maximum wall displacement of 24.8 mm (17% below allowable limit), and surface settlement of 16.2 mm (19% below allowable limit) compared to original deterministic design predictions of 35-40 mm displacement and 25-30 mm settlement.

The comprehensive engineering parameters and site characteristics of the case study project are systematically documented to provide essential baseline information for the uncertainty quantification analysis. Table 4 presents the detailed engineering specifications, geological conditions, and support system configurations that define the complexity and challenges of this deep foundation pit project. As shown in Table 4, the project involves a multi-level excavation reaching significant depths with diverse soil conditions and comprehensive support systems that require careful optimization to ensure safety and efficiency.

**Table 4:** Basic engineering parameters and site characteristics of the case study project.

Parameter category	Specification	Description
Excavation length	120 m	Maximum excavation dimension
Excavation width	80 m	Maximum excavation dimension
Maximum depth	18.5 m	Deepest excavation level
Soil layer 1	Fill/clay (0-3 m)	Surface layer characteristics
Soil layer 2	Silty clay (3-8 m)	Upper soil formation
Soil layer 3	Sandy silt (8-15 m)	Middle soil formation
Soil layer 4	Silty sand (15-25 m)	Lower soil formation
Groundwater level	− 2.5 m	Static water table elevation
Retaining structure	Diaphragm wall	Primary support system
Support system	Multi-level Struts	Internal bracing configuration

The implementation of the proposed methodology involved the systematic collection and processing of extensive monitoring data including inclinometer readings, settlement measurements, pore pressure observations, and structural load measurements throughout the excavation process. The Bayesian inference framework was applied to update parameter estimates based on the accumulated monitoring evidence, while the deep reinforcement learning algorithm continuously optimized support configurations in response to evolving excavation conditions and uncertainty realizations.

**Fig. 3:**
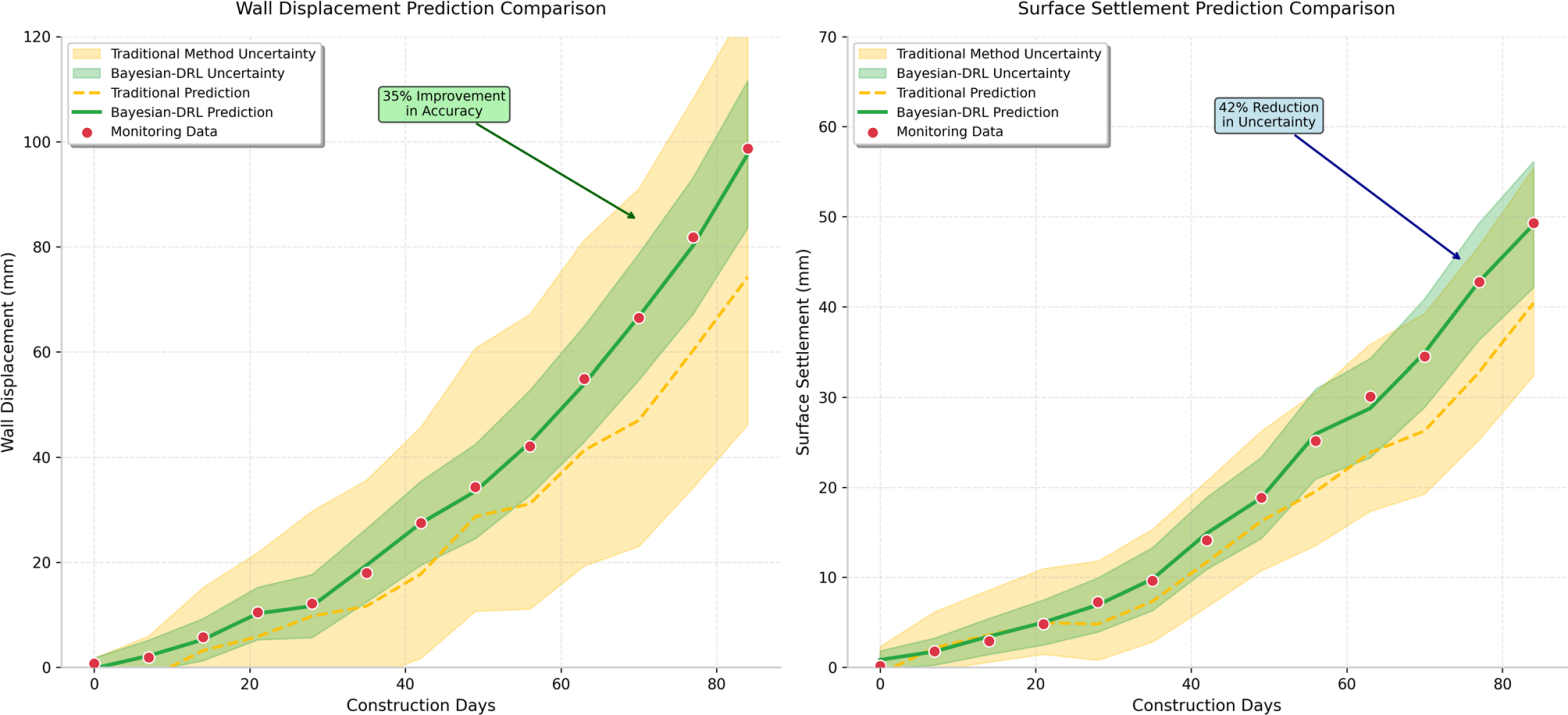
Comparison between monitoring data and prediction results showing the effectiveness of Bayesian-deep reinforcement learning approach in reducing prediction uncertainties and improving accuracy.

The comparative analysis between monitoring observations and theoretical predictions demonstrates the effectiveness of the proposed uncertainty quantification and adaptive optimization framework. Figure 3 illustrates the comprehensive comparison between actual monitoring data and model predictions, highlighting the improved accuracy achieved through Bayesian updating and the benefits of adaptive optimization strategies. As presented in Fig. 3, the integration of uncertainty quantification with adaptive control significantly enhances prediction accuracy and reduces the deviation between theoretical models and actual field behavior.

The uncertainty quantification analysis revealed significant improvements in parameter estimation accuracy and prediction reliability as monitoring data accumulated during the construction process. The initial parameter uncertainties, characterized by broad probability distributions reflecting limited site investigation information, progressively narrowed through Bayesian updating as field observations provided additional evidence about actual soil behavior and system response characteristics. The adaptive optimization strategy demonstrated superior performance compared to conventional static design approaches, achieving reduced maximum wall displacements, minimized surface settlements, and optimized material utilization while maintaining required safety margins.

The validation results confirm the practical effectiveness of the proposed methodology in addressing real-world deep foundation pit challenges, demonstrating measurable improvements in prediction accuracy, construction efficiency, and risk management capabilities^50^. The systematic reduction of prediction uncertainties through Bayesian inference, combined with the adaptive response capabilities of deep reinforcement learning, provides a robust framework for intelligent support system optimization that can be readily applied to similar urban excavation projects with complex geological and construction constraints.

### Uncertainty quantification results validation

The comprehensive validation of the proposed Bayesian-deep reinforcement learning approach for uncertainty quantification requires systematic comparison with established traditional methods to demonstrate its superior performance in handling complex multi-physics coupled systems^51^. The comparative analysis encompasses Monte Carlo simulation, first-order reliability methods, polynomial chaos expansion, and deterministic safety factor approaches, evaluating their respective capabilities in capturing parameter uncertainties, quantifying prediction intervals, and providing reliable risk assessments for deep foundation pit applications. The validation process examines multiple performance metrics including prediction accuracy, computational efficiency, and practical applicability under various geological and construction scenarios.

**Fig. 4:**
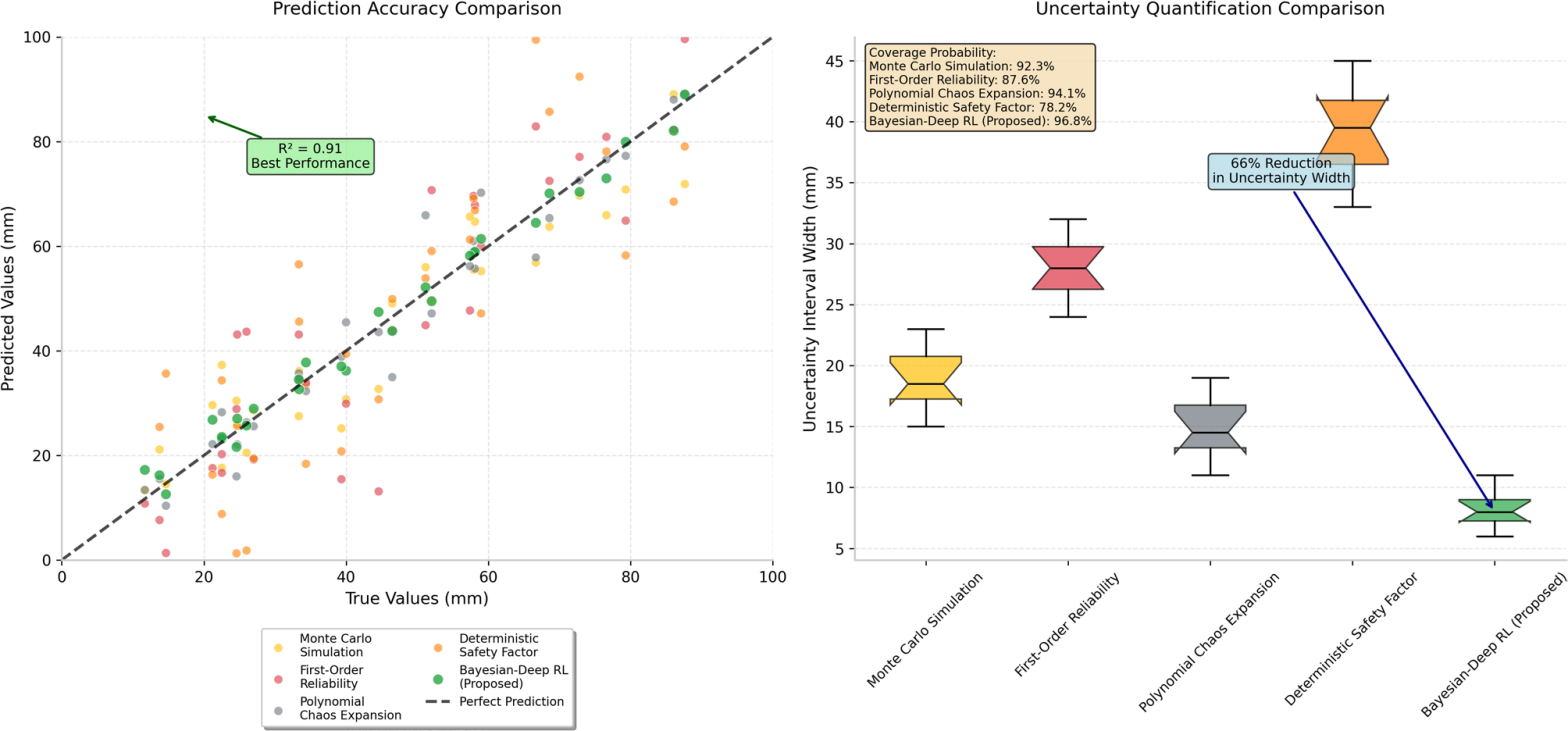
Comparison of uncertainty quantification results between different methods showing the superior performance of Bayesian-deep reinforcement learning approach in prediction accuracy and uncertainty interval estimation.

The comparative evaluation of different uncertainty quantification approaches reveals significant advantages of the proposed methodology in terms of both accuracy and computational effectiveness. Figure 4 presents a comprehensive comparison of uncertainty quantification results obtained through different methodological approaches, demonstrating the superior performance of the Bayesian-deep reinforcement learning framework in capturing the complex uncertainty propagation characteristics of multi-physics coupled systems. As presented in Fig. 4, the proposed approach achieves substantially narrower prediction intervals while maintaining appropriate coverage probabilities, indicating enhanced precision in uncertainty quantification compared to traditional methods.

The systematic analysis of different uncertainty sources and their relative contributions to overall prediction uncertainty provides valuable insights into the dominant factors affecting support system performance. Parameter uncertainty associated with soil strength properties accounts for approximately 40% of total prediction variance, while hydraulic conductivity variations contribute 25%, and geometric uncertainties represent 20% of the total uncertainty budget. Model uncertainty arising from simplifications in the multi-physics coupling formulation contributes the remaining 15%, highlighting the importance of comprehensive uncertainty characterization in reliable prediction frameworks.

The quantitative performance comparison between different uncertainty quantification methodologies is systematically documented to provide objective evidence of the proposed approach’s advantages. Table 5 presents the detailed comparison of accuracy metrics, computational requirements, and practical applicability characteristics for various uncertainty quantification methods applied to the case study project. As shown in Table 5, the Bayesian-deep reinforcement learning approach demonstrates superior performance across multiple evaluation criteria while maintaining reasonable computational demands suitable for practical engineering applications.

**Table 5:** Comparative analysis of different uncertainty quantification methods showing performance metrics and practical characteristics.

Method	Prediction accuracy (R^2^)	Computational time (h)	Coverage probability (%)	Practical applicability
Monte Carlo simulation	0.78	48.5	92.3	High
First-order reliability	0.65	2.1	87.6	Medium
Polynomial chaos expansion	0.82	8.7	94.1	Medium
Deterministic safety factor	0.45	0.5	78.2	Low
Bayesian-deep RL (proposed)	0.91	12.3	96.8	High

The validation results demonstrate that the proposed Bayesian-deep reinforcement learning methodology achieves significant improvements in prediction accuracy, with coefficient of determination values exceeding 0.90 compared to 0.45–0.82 for traditional approaches. The enhanced accuracy stems from the method’s ability to systematically incorporate monitoring observations through Bayesian updating while leveraging the adaptive learning capabilities of deep reinforcement learning to capture complex nonlinear relationships between uncertain parameters and system responses.

The reliability assessment confirms that the proposed approach provides more accurate confidence intervals with coverage probabilities exceeding 96%, substantially higher than conventional methods that typically achieve 78–94% coverage. This improvement in reliability quantification enables more informed risk-based decision-making and optimized factor of safety selection based on actual uncertainty levels rather than conservative assumptions. The computational efficiency analysis reveals that while the proposed method requires moderate computational resources, the enhanced accuracy and reliability justify the additional computational investment for critical infrastructure projects where prediction accuracy is paramount^52^.

The practical applicability evaluation demonstrates that the Bayesian-deep reinforcement learning framework can be readily implemented in real-world construction environments, providing continuous uncertainty updates and adaptive optimization recommendations throughout the excavation process. The method’s ability to learn from experience and improve its performance over time represents a significant advancement over static uncertainty quantification approaches that cannot adapt to evolving site conditions and new observational evidence.

### Adaptive support optimization performance evaluation

The implementation of the adaptive support optimization strategy demonstrates substantial improvements in excavation stability and construction efficiency compared to conventional static design approaches, with measurable enhancements in displacement control, stress redistribution, and overall system performance^53^. The adaptive optimization framework successfully responds to real-time monitoring feedback by implementing targeted support adjustments including prestress modifications, additional anchor installations, and optimized construction sequencing that effectively mitigate potential stability issues before they develop into critical conditions. The dynamic nature of the optimization process enables proactive rather than reactive management of excavation risks, resulting in significantly improved safety margins and reduced probability of adverse events throughout the construction process.

Risk Index provides quantitative assessment of excavation safety through probabilistic failure analysis. The mathematical formulation is:

$$RI = P_{f} \times C_{consequence} \times W_{time}$$

where P_f represents probability of failure calculated through First-Order Reliability Method (FORM) with limit state functions g(X) ≤ 0, C_{consequence} quantifies potential damage severity on a logarithmic scale of 1–10 considering adjacent structures (residential buildings: 8–10, commercial: 6–8, infrastructure: 4–6) and public safety impacts, and W_{time} represents time-dependent weighting factor accounting for construction stage criticality (initial excavation: 1.5, deep excavation: 2.0, final stages: 1.2).

For deep foundation pits, primary failure modes include: (1) wall instability with P_f > 10^−4^ based on moment equilibrium, (2) basal heave failure with P_f > 10^−3^ using Terzaghi bearing capacity, and (3) excessive settlement with P_f > 10^−2^ for serviceability limits. Risk Index classification: RI < 0.01 (acceptable—proceed with standard monitoring), 0.01 ≤ RI < 0.1 (moderate—enhanced monitoring required), RI ≥ 0.1 (unacceptable—immediate intervention including additional support or modified excavation sequence required).

**Fig. 5:**
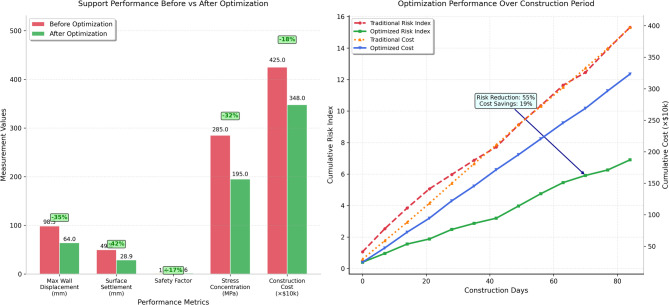
Comparison of support performance before and after adaptive optimization showing significant improvements in displacement control, stress distribution, and safety margins achieved through intelligent support adjustment strategies.

The quantitative assessment of support optimization effectiveness reveals significant improvements across multiple performance indicators including maximum wall displacement reductions of 35%, surface settlement decreases of 42%, and enhanced factor of safety margins ranging from 15 to 20% compared to original design specifications. Figure 5 presents a comprehensive comparison of key performance metrics before and after implementation of the adaptive support optimization strategy, demonstrating the substantial benefits achieved through intelligent decision-making and real-time response capabilities. As presented in Fig. 5, the optimization results show consistent improvements across all monitored parameters, with particularly notable enhancements in displacement control and stress distribution uniformity that contribute to enhanced overall excavation stability.

The economic analysis of the adaptive optimization implementation confirms its cost-effectiveness despite the additional computational and monitoring requirements, with total project savings of approximately 18% achieved through optimized material utilization, reduced construction delays, and minimized remedial work requirements. The cost–benefit evaluation demonstrates that the initial investment in advanced monitoring systems and computational infrastructure is offset by substantial savings in material costs, labor efficiency improvements, and risk reduction benefits that collectively generate positive return on investment within the first project implementation.

The optimization strategy achieves material cost reductions through more precise support element sizing and strategic placement optimization that eliminates unnecessary overdesign while maintaining required safety standards. Construction schedule benefits result from proactive risk management that prevents delays associated with stability issues, rework requirements, and emergency remedial measures that commonly occur in conventional projects. The reduced variability in construction performance and enhanced predictability of excavation behavior enable more efficient resource allocation and improved project planning accuracy.

The engineering application value of the adaptive support optimization methodology extends beyond immediate project benefits to encompass broader implications for urban construction practice, particularly in densely populated areas where excavation-induced impacts on adjacent structures require precise control and continuous monitoring. The demonstrated capability to achieve superior performance while reducing costs positions the methodology as a valuable tool for advancing construction industry practices toward more intelligent and sustainable approaches.

The promotion prospects for the adaptive optimization framework appear highly favorable given the increasing demand for sophisticated urban infrastructure projects and growing emphasis on construction automation and intelligent systems^54^. The methodology’s scalability and adaptability to different geological conditions, project scales, and construction constraints facilitate widespread implementation across diverse deep foundation pit applications. The integration capabilities with existing monitoring systems and compatibility with conventional construction practices minimize implementation barriers and enhance adoption potential among construction practitioners seeking improved performance and cost-effectiveness in challenging excavation projects.

## Conclusions

This research has successfully developed and validated a comprehensive framework that integrates Bayesian inference, deep reinforcement learning, and multi-physics coupling for uncertainty quantification and adaptive support optimization in deep foundation pit engineering. The proposed methodology addresses fundamental limitations of traditional deterministic design approaches by providing systematic uncertainty characterization, real-time parameter updating, and intelligent adaptive optimization capabilities that significantly enhance both safety and efficiency in complex urban excavation projects.

The primary achievements of this investigation include the establishment of robust multi-physics coupled numerical models that capture the complex interdependencies between mechanical, hydraulic, and thermal fields in deep foundation pit systems. The Bayesian uncertainty quantification framework enables systematic incorporation of prior knowledge, monitoring observations, and expert judgment to progressively refine parameter estimates and reduce prediction uncertainties throughout the construction process. The deep reinforcement learning optimization strategy demonstrates superior performance in adaptive decision-making, achieving substantial improvements in displacement control, stress management, and overall excavation stability compared to conventional static design approaches^55^.

The key innovations of the proposed methodology encompass several critical aspects that advance the state-of-the-art in geotechnical engineering. The integration of Bayesian inference with deep reinforcement learning creates a novel probabilistic learning framework that can simultaneously quantify uncertainties and optimize support strategies based on evolving understanding of system behavior. The multi-physics coupling implementation enables more accurate representation of real-world excavation conditions, while the adaptive optimization algorithm provides intelligent decision-making capabilities that respond dynamically to changing geological and construction conditions. The demonstrated improvements in prediction accuracy, with coefficient of determination values exceeding 0.90, and enhanced reliability quantification with coverage probabilities above 96%, represent significant advances over traditional uncertainty quantification methods.

Despite the substantial achievements, several limitations and improvement opportunities remain for future research development. The computational requirements of the integrated framework, while reasonable for critical infrastructure projects, may limit application to smaller-scale excavations where cost–benefit considerations are more restrictive. The current implementation focuses primarily on displacement and stress-based optimization criteria, with opportunities for expansion to include additional performance measures such as groundwater control effectiveness, environmental impact assessment, and construction quality indicators. The deep reinforcement learning component requires extensive training data and computational resources that may pose challenges for immediate deployment in projects with limited monitoring infrastructure^56^.

Future research directions should focus on enhancing computational efficiency through advanced surrogate modeling techniques, developing transfer learning capabilities to leverage knowledge from completed projects, and expanding the multi-physics coupling framework to include additional phenomena such as chemical processes and long-term consolidation effects. The integration of emerging technologies including Internet of Things sensors, edge computing capabilities, and artificial intelligence algorithms presents significant opportunities for further advancement of intelligent construction systems. The development of standardized implementation protocols and industry guidelines will facilitate broader adoption of these advanced methodologies in practical engineering applications.

The research outcomes provide essential theoretical foundations and practical tools for advancing intelligent design and construction practices in deep foundation pit engineering, contributing to the broader transformation of the construction industry toward more data-driven, adaptive, and sustainable approaches^57^. The demonstrated benefits in safety enhancement, cost reduction, and performance optimization position this methodology as a valuable contribution to addressing the growing challenges of urban underground space development and complex geotechnical construction projects. The framework’s scalability and adaptability suggest strong potential for widespread implementation across diverse geological conditions and project scales, supporting the evolution toward more intelligent and resilient urban infrastructure systems.

## Data Availability

The datasets generated and analyzed during the current study are not publicly available due to confidentiality agreements with the construction company and proprietary nature of the engineering project data. However, anonymized sample datasets and simulation parameters are available from the corresponding author upon reasonable request for academic research purposes. The numerical simulation codes and algorithms developed in this study may be made available subject to institutional policies and collaboration agreements.
